# In relapsed or refractory diffuse large B‐cell lymphoma, CD19 expression by immunohistochemistry alone is not a predictor of response to loncastuximab tesirine

**DOI:** 10.1002/jha2.816

**Published:** 2023-11-30

**Authors:** Paolo F. Caimi, Mehdi Hamadani, Carmelo Carlo‐Stella, Masoud Nickaeen, Eric Jordie, Kiersten Utsey, Tim Knab, Francesca Zammarchi, Danilo Cucchi, Serafino Pantano, Karin Havenith, Ying Wang, Joseph Boni

**Affiliations:** ^1^ Blood and Marrow Transplant Program Taussig Cancer Institute Cleveland Clinic Foundation Cleveland Ohio USA; ^2^ Division of Hematology and Oncology BMT and Cellular Therapy Program Medical College of Wisconsin Milwaukee Wisconsin USA; ^3^ Department of Biomedical Sciences Humanitas University and Department of Hematology and Oncology, IRCCS Humanitas Research Hospital Milan Italy; ^4^ Metrum Research Group Simsbury Connecticut USA; ^5^ ADC Therapeutics (United Kingdom) Ltd London UK; ^6^ ADC Therapeutics SA Épalinges Switzerland; ^7^ ADC Therapeutics Murray Hill New Jersey USA

**Keywords:** CD19, diffuse large B‐cell lymphoma, immunohistochemistry, loncastuximab tesirine, quantitative systems pharmacology

## Abstract

CD19‐targeting treatments have shown promise in relapsed/refractory (R/R) diffuse large B‐cell lymphoma (DLBCL). Loncastuximab tesirine (loncastuximab tesirine‐lpyl [Lonca]) is a CD19‐targeting antibody‐drug conjugate indicated for R/R DLBCL after at least two systemic treatments. CD19 expression was evaluated in patients receiving Lonca in the LOTIS‐2 clinical trial with available tissue samples obtained after last systemic therapy/before Lonca treatment. Lonca cytotoxicity was evaluated in a panel of six lymphoma cell lines with various CD19 expression levels. Quantitative systems pharmacology (QSP) modelling was used to predict Lonca responses. Lonca responses were seen in patients across all CD19 expression levels, including patients with low/no detectable CD19 expression and H‐scores at baseline. Similarly, Lonca induced cytotoxicity in cell lines with different levels of CD19 expression, including one with very low expression. QSP modelling predicted that CD19 expression by immunohistochemistry alone does not predict Lonca response, whereas inclusion of CD19 surface density improved response prediction. Virtual patients responded to Lonca with estimated CD19 as low as 1000 molecules/cell of CD19, normally below the immunohistochemistry detection level. We found Lonca is an effective treatment for R/R DLBCL regardless of CD19 expression by immunohistochemistry. These results provide the basis for future studies addressing CD19‐targeted agent sequencing.

## Graphical abstract

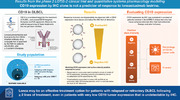

## 1 INTRODUCTION

Diffuse large B‐cell lymphoma (DLBCL) is the most common aggressive subtype of non‐Hodgkin lymphoma, and approximately 40% of patients have refractory disease or relapse after first‐line therapy [1, 2, 3, 4]. CD19 is a type I transmembrane glycoprotein that plays a critical role in the regulation of signalling pathways responsible for B‐cell proliferation and differentiation [5]. In normal human cells, CD19 is expressed through pre–B‐cell and mature B‐cell differentiation until it is downregulated during terminal differentiation into plasma cells [6]. Expression of CD19 is maintained in haematological B‐cell malignancies, including DLBCL, follicular lymphoma and mantle cell lymphoma, and the majority of B‐cell malignancies express CD19 at normal‐to‐high levels [7]. For these reasons, CD19 is a clinically validated target for the treatment of B‐cell malignancies [8].

The National Comprehensive Cancer Network guidelines now recommend repeat biopsy in patients with DLBCL with no response or progressive disease (PD) after first‐line therapy to confirm positron emission tomography positivity prior to additional therapy [9]. When these biopsies are available, they also present an opportunity to evaluate CD19 expression to inform treatment decisions. However, immunohistochemistry (IHC) is not reliable enough to distinguish between intracellular and membranous antigen expression, which is needed for CD19‐targeted therapies [10].

Loncastuximab tesirine (loncastuximab tesirine‐lpyl [Lonca]) is an antibody‐drug conjugate (ADC) comprising a humanised anti‐CD19 antibody conjugated to a pyrrolobenzodiazepine dimer cytotoxin, SG3199, and is indicated for patients with R/R DLBCL, DLBCL arising from low‐grade lymphoma and high‐grade B‐cell lymphoma after at least two systemic treatments [11, 12, 13, 14]. Upon binding to CD19, Lonca is internalised, and SG3199 is released and binds to the DNA minor groove, forming highly cytotoxic DNA interstrand crosslinks [12]. The safety and efficacy of Lonca have been studied in phase 1 (LOTIS‐1; NCT02669017) and phase 2 (LOTIS‐2; NCT03589469) clinical trials, which have demonstrated substantial single‐agent antitumour activity and durable responses with an acceptable safety profile [15, 16, 17].

Results from exploratory analyses of the LOTIS‐1 and LOTIS‐2 clinical trials suggest that previous CD19‐targeted chimeric antigen receptor T‐cell (CAR‐T) therapy does not preclude response to Lonca; additionally, patients who previously received Lonca experienced a response to subsequent CAR‐T therapy [18, 19]. However, the value of CD19 expression alone in predicting response to CD19‐directed therapies, such as Lonca or CD19‐directed CAR‐T, is unclear. Here, we present results from an exploratory analysis of the LOTIS‐2 clinical trial and quantitative systems pharmacology (QSP) modelling that evaluated the relationship between CD19 expression and response to Lonca.

## 2 METHODS

### 2.1 Study design

Archival tumour tissue samples were obtained from a subset of patients who had biopsies collected after their last anticancer systemic therapy and before their enrolment in the multicentre, open‐label, single‐arm, phase 2 LOTIS‐2 study (NCT03589469) and also when these tissue samples were available for evaluation (Table S1). The LOTIS‐2 study design and methodology have been previously described [15]. In brief, eligible patients included adults (aged ≥18 years) with R/R DLBCL after two or more multiagent systemic treatments who had measurable disease and an Eastern Cooperative Oncology Group performance status of 0–2. Previous treatment with a CD19‐directed therapy, including CD19‐directed CAR‐T therapy, was permitted if CD19 expression was confirmed by biopsy prior to treatment with Lonca.

In LOTIS‐2, eligible patients received Lonca intravenously on day 1 of each 21‐day cycle at 150 μg/kg for two cycles, then 75 μg/kg thereafter, for up to 1 year or until disease relapse or progression; unacceptable toxicity; death; major protocol deviation; pregnancy; or patient, investigator or sponsor decision. Patients could continue treatment beyond 1 year upon agreement of the sponsor. The primary endpoint was the overall response rate, per the 2014 Lugano classification [20], determined by an independent central review board (Table S2). Lonca responders were defined as patients with a best overall response of a complete response (CR) or partial response (PR). Nonresponders included all other response categories, including PD, stable disease (SD) and not evaluable (NE).

LOTIS‐2 was conducted in accordance with the Declaration of Helsinki and the International Conference on Harmonisation Guidelines for Good Clinical Practice. All patients provided written informed consent. The study protocols and amendments were approved by all relevant ethics committees.

### 2.2 CD19 immunohistochemistry

CD19 expression was determined by IHC in a subgroup of patients enrolled in the LOTIS‐2 clinical study, with available tissue samples obtained after their last systemic anticancer therapy and prior to Lonca treatment. Patients who had received previous CD19‐directed therapy must have had a biopsy confirming CD19 protein expression after completion of the CD19‐directed therapy. For available biopsy samples, 4‐ to 5‐μm thick tissue sections were deparaffinized on the BenchMark ULTRA platform (Ventana Medical Systems, Roche Group, Tucson, Arizona, USA) using the EZ prep solution. The standard antigen retrieval method was performed by CC1 (prediluted; tris‐EDTA buffer pH of 7.8 for 64 min at 95°C) antigen retrieval solution (Ventana Medical Systems, Roche Group, Tucson, Arizona, USA) on the BenchMark ULTRA automated slide stainer (Ventana) for 64 min at 100°C (default temperature on ULTRA). Primary antibody incubation was performed using a mouse monoclonal CD19 (clone LE‐CD19; Dako, Cat# M7296) at a 1:20 dilution for 32 min at 37°C, followed by visualization with the OptiView DAB IHC Detection Kit (Ventana) for 16 min. The specimens were counterstained with Hematoxylin II and Bluing Reagent (Ventana). Each IHC run contained positive and negative tissue controls and a negative reagent control (Ventana, Cat# 760–2014).

CD19 expression was assessed by semiquantitative scoring of both the percentage of positive tumour cells and histoscore (H‐score), which is a semiquantitative assessment based on the percentage of CD19‐positive cells and staining intensity. The H‐score is a report of the percentage of cells within one of the following categories: negative (0), weakly (1+), moderately (2+), and strongly (3+) stained membranes. An H‐score with a potential range of 0 to 300 was calculated as follows:

$$\begin{array}{ccc} H-score & & =\left(1\times \%\;weakly\;stained\;cells\right)\\ & & \;+\left(2\times \%\;moderately\;stained\;cells\right)\\ & & \;+\left(3\times \%\;strongly\;stained\;cells\right) \end{array}$$

### 2.3 Quantification of CD19 expression and Lonca in vitro cytotoxicity in a panel of lymphoma cell lines

The in vitro cytotoxicity of Lonca was evaluated in a panel of six B‐cell non‐Hodgkin lymphoma cell lines, including OCI‐Ly3, SU‐DHL‐2, TMD8, SU‐DHL‐16, SU‐DHL‐4, and MEC‐1. Cell lines were obtained from ATCC or DSMZ, and cell growth conditions have been previously described by Zammarchi et al. [11]. The cytotoxicity of Lonca was determined by the CellTiter 96 Aqueous One Solution Cell Proliferation Assay (MTS assay) (Promega, Madison, Wisconsin, USA). The half maximal inhibitory concentration (IC50) values were determined using GraphPad Prism 9.4.1 software.

The CD19 cell surface density in lymphoma cell lines was determined by flow cytometry using Bangs Laboratory Quantum MESF beads, according to the manufacturer's instructions. The CD19 IHC analysis in lymphoma cell lines was performed following the same protocol and scoring methods used for the clinical samples.

### 2.4 QSP modelling

Virtual patient simulations were performed using multifactorial QSP modelling to predict response to Lonca. Virtual patients comprise a collection of mathematic models, parameters and associated discrete variability in those parameters, which are estimated from clinical and preclinical data (Table S3). Patient‐specific biological and pharmacological factors incorporated in this model included initial tumour size and location, patient body weight, the rate of Lonca‐induced tumour cell death, the rate of Lonca cellular internalization, the rate of payload diffusion out of cells, the growth rate of tumour cells, and CD19 expression level and surface density (molecules/cell) from pretreatment tumour biopsies. Tissue volumes and blood flow rates of the physiologically based pharmacokinetic submodel were allometrically scaled based on body weight:

$$Vol=Vol_{ref}\cdot \left(BW/BW_{ref}\right)\;\mathrm{and}\;Flow=Flow_{ref}\cdot {\left(BW/BW_{ref}\right)}^{0.75}$$

Patient pharmacokinetic data and tumour size during the first 100 days (after the first dose) were used to evaluate model predictions. Effects of other important intrinsic factors, including but not limited to disease phenotype (double‐hit and triple‐hit DLBCL) and hypoalbuminemia (as neonatal FcRn receptor expression), were also explored. Response to Lonca was determined by comparing the area under the curve (AUC) of the tumour volume dynamics against the AUC of a stable disease scenario (initial tumour volume multiplied by the total simulation duration).

### 2.5 Statistical analysis

Data are represented as a function of the best overall response by independent assessment (CR, PR, SD and PD). The analysis to compare box plots was performed using the stat_compare_means function in the ggpubr library version 0.4.0, as implemented in R version 4.0.3.

### 2.6 Data‐sharing statement

The study protocol was published previously [15]. Summary data for NCT03589469 are posted at ClinicalTrials.gov, as required. Proposals requesting original participant data collected for the study can be sent to Joe.Boni@adctherapeutics.com.

## 3 RESULTS

### 3.1 Patients

**FIGURE 1:**
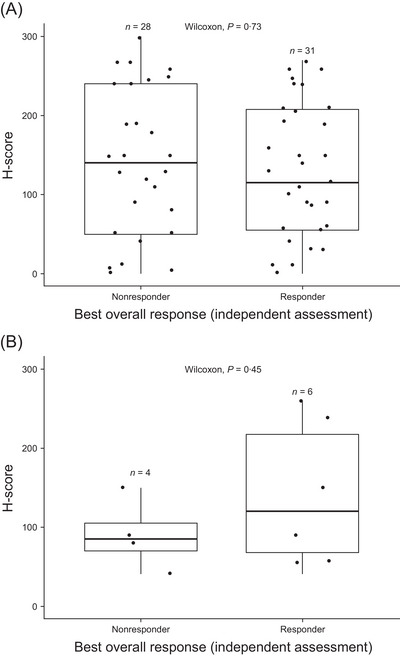
Baseline tumour CD19‐positive H‐score by response to Lonca in LOTIS‐2 assessed by an independent assessment. (A) IHC analysis of biopsy samples from patients (*n* = 59; 28 nonresponders and 31 responders) and (B) from patients after CAR‐T therapy (*n* = 10; four nonresponders and six responders). H‐score (scale, 0–300) represents the percentage of negative (0), weakly (1+), moderately (2+) and strongly (3+) stained membranes cells calculated as follows: H‐score = (1 × % weakly stained cells) + (2 × % moderately stained cells) + (3 × % strongly stained cells). The middle line denotes the median. The box denotes the 25th and 75th percentiles of observed data. CAR‐T, chimeric antigen receptor T‐cell; H‐score, histoscore; IHC, immunohistochemistry; Lonca, loncastuximab tesirine‐lpyl.

The current study included patients enrolled in the LOTIS‐2 study with biopsies that were collected after the last systemic therapy and before Lonca treatment (*n* = 59). Of these 59 patients, 28 patients were nonresponders, and 31 were responders (Figure 1A). Ten patients received CD19‐directed CAR‐T therapy before Lonca treatment, including four Lonca nonresponders and six responders (Figure 1B). The median time from biopsy to Lonca treatment for these 59 patients was 18·0 days (range, 1·5–185 days).

### 3.2 Response to Lonca was observed regardless of tumour CD19 expression in patient baseline samples

Clinical responses to Lonca treatment were observed in patients regardless of baseline CD19 expression based on IHC. This included patients with low or no detectable CD19 expression at baseline who had an extremely low H‐score or H‐score of 0 (Figure 1A). Responses to Lonca were also observed in patients who received CD19‐directed CAR‐T therapy (*n* = 10), including those with reportable H‐score values (Figure 1B).

Nine patients had a CD19 H‐score ≤10, 4/9 (44%) exhibited a response to Lonca (all were PR), and the durations of response were 47, 50, 81, and 282 days. None of the patients with an H‐score ≤10 who exhibited a response had received CD19‐directed CAR‐T therapy. Four patients had a CD19 H‐score ≤5, 1/4 (25%) exhibited a response to Lonca (which was a PR), and the duration of response was 282 days. Fifty patients had a CD19 H‐score >10, and 27 of 50 (54%) exhibited a response to Lonca. No statistically significant differences in H‐scores were apparent among the response categories (CR, PR, SD, PD or NE), as depicted in Figure S1.

### 3.3 Lonca cytotoxicity was observed in B‐cell non‐Hodgkin lymphoma cell lines across different levels of CD19 expression

**FIGURE 2:**
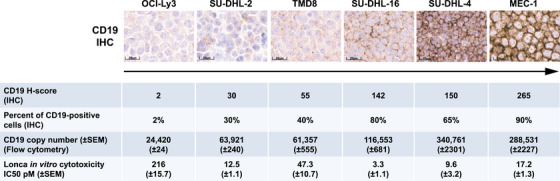
Quantification of CD19 expression (IHC and flow cytometry) and Lonca in vitro cytotoxicity in a panel of six B‐cell non‐Hodgkin lymphoma cell lines. Images are at 20× magnification. H‐score, histoscore; IC50, half maximal inhibitory concentration; IHC, immunohistochemistry; Lonca, loncastuximab tesirine‐lpyl; SEM, standard error of the mean.

Lonca in vitro cytotoxicity was assessed in a panel of six B‐cell non‐Hodgkin lymphoma (NHL) cell lines. CD19 expression in the six cell lines was evaluated in two ways: by IHC, following the same protocol and semiquantitative scoring method used for the clinical samples, and by quantitative flow cytometry, allowing the determination of the CD19 cell surface copy number. The percentage of CD19‐positive cells ranged from 2% (OCI‐Ly3) to 90% (MEC‐1) (H‐score from 2 to 265) as determined by IHC, which corresponded to CD19 copy numbers ranging from 24 420 (± 24) (OCI‐Ly3) to 340 761 (± 2301) (SU‐DHL‐4) by flow cytometry (Figure 2).

Lonca showed potent cytotoxicity (IC50 within the pM range) across the panel of six cell lines with IC50 values ranging from 3·3 (± 1·1) pM in the SU‐DHL‐16 cell line (CD19 H‐score of 142; 80% CD19‐positive cells) to 216 (± 15·7) pM in OCI‐Ly3 (CD19 H‐score of 2; 2% CD19‐positive cells) (Figure 2).

### 3.4 CD19 expression by IHC and tumour cell surface density improve model predictions of response to Lonca

**FIGURE 3:**
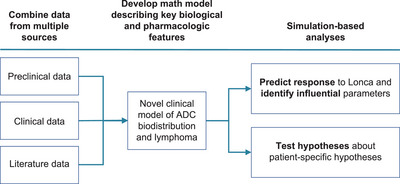
Quantitative systems pharmacology model schematic. ADC, antibody‐drug conjugate; Lonca, loncastuximab tesirine‐lpyl.

**FIGURE 4:**
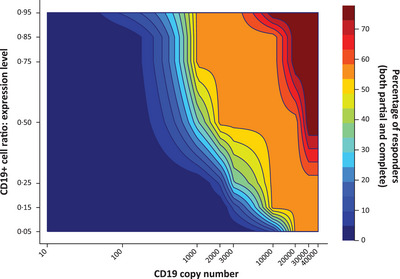
Lonca heat map profile of CD19‐positive cell ratio of expression versus CD19 surface density and response. Lonca, loncastuximab tesirine lpyl.

**FIGURE 5:**
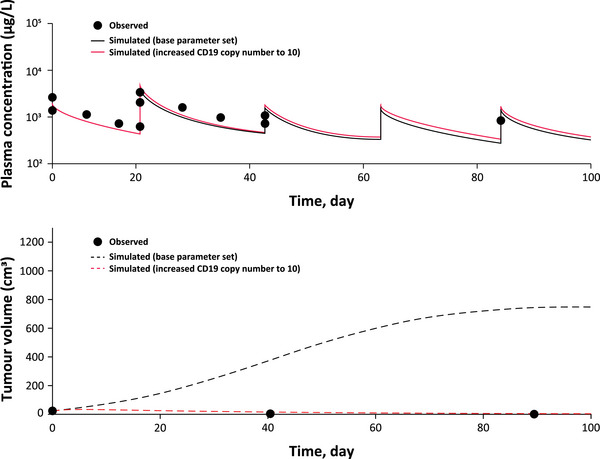
Responses were seen in patients across all levels of CD19 expression, including patients with undetectable CD19 expression. The example patient had a body weight of 54 kg; biopsy showed undetectable CD19 expression by IHC (black circles in the upper graph), but a complete response to Lonca was observed (black circles in the lower graph). QSP model indicated that undetectable CD19 surface density (solid red line in the upper graph) was sufficient for response (dotted red line in the lower graph). IHC, immunohistochemistry; Lonca, loncastuximab tesirine lpyl; QSP, quantitative systems pharmacology.

A total of 567 virtual patient simulations were performed by scanning CD19‐positive expression levels, CD19 antigen surface densities per tumour cells, the initial tumour mass and the tumour location using Lonca dosing from the LOTIS‐2 study (150 μg/kg every 3 weeks for the first two cycles, followed by 75 μg/kg for subsequent cycles) (Figure 3; Figure S2). Virtual patient simulations performed using the QSP model predicted possible disease response to Lonca with CD19 tumour cell surface densities as low as 1000 molecules/cell (Figures 4 and 5). QSP modelling indicated that the faster growth rate of double‐hit lymphomas might explain the observed lack of response to Lonca, despite high CD19 positivity, as measured by IHC (Figure S3). Patients with hypoalbuminemia had enhanced clearance and reduced Lonca exposure, as assessed through a reduction in FcRn expression (Figure S4).

## 4 DISCUSSION

CD19 is a clinically validated treatment target in DLBCL, and it is of increasing importance for R/R disease owing to the availability of a number of CD19‐directed therapeutic options. However, clinical data to guide treatment sequencing in this setting are still scarce. Lonca is a CD19‐targeting antibody‐drug conjugate that demonstrated durable efficacy and an acceptable safety profile in heavily pretreated patients with R/R DLBCL in the LOTIS‐2 study [15]. Exploratory analyses of the LOTIS‐1 and LOTIS‐2 clinical studies suggest that previous CD19‐directed CAR‐T therapy does not preclude response to Lonca and that previous treatment with Lonca does not preclude subsequent responses to subsequent CD19‐directed CAR‐T therapy, but the correlation between CD19 expression and disease response in patients treated with Lonca was previously unknown [18, 19].

In this LOTIS‐2 exploratory analysis, clinical response to Lonca was observed in patients with R/R DLBCL with very low or undetectable CD19 tumour expression as measured by IHC. Semiquantitative scoring of CD19 expression by IHC (H‐score) did not correlate with response to Lonca. Further, Lonca showed potent cytotoxicity in a panel of six B‐cell NHL human cell lines with different levels of CD19 expression and CD19 copy numbers, as measured by IHC and flow cytometry, respectively. Notably, Lonca‐induced cytotoxicity was observed in cell lines with low CD19 IHC expression, including a cell line in which 98% of cells were CD19 negative when evaluated by IHC.

It is of relevance that negativity by IHC indicates CD19 levels are below the detection limit of the assay, but cells could still have CD19 expression that is not detectable by IHC. For example, Lonca induced cytotoxicity in OCI‐Ly3 cells in which 2% of tumour cells were CD19 positive by IHC but had ∼24,000 CD19 copy numbers by flow cytometry. Although IHC is a common technique used to evaluate CD19 expression, the results presented here suggest that IHC might not be a sensitive enough assay to evaluate CD19 expression. Therefore, a more sensitive assay, such as flow cytometry, should be considered to evaluate CD19 expression and potentially predict response to CD19‐directed agents [9, 20, 21]. The National Comprehensive Cancer Network guidelines for B‐cell lymphomas recommend flow cytometry to evaluate CD19 expression in patients who relapse or have refractory disease [9].

Experimental data indicated that 24,000 copies/cell in B‐cell NHL cell lines was sufficient for Lonca‐induced cytotoxicity, and QSP modelling predicted that response to Lonca may be achieved in patients with CD19 expression as low as 1000 molecules/cell. This is below the recent threshold of 3000 molecules/cell identified as a cutoff for CD19 positivity [22, 23]. Similar to the results presented here, CD19‐targeting axicabtagene ciloleucel CAR‐T therapy responses were observed in patients with relapsed/refractory B‐cell NHL who had undetectable levels of CD19 in the phase 2 ZUMA‐1 clinical trial (NCT02348216), suggesting that CD19 expression levels did not impact response rates to or durable responses after axicabtagene ciloleucel; however, further investigations are necessary to understand better if and how risk of relapse could be increased in patients with a low CD19 copy number [20, 22, 24]. Although the optimal sequencing of CD19‐directed therapies has not been clarified, the results presented here, as well as those from ZUMA‐1, suggest that CD19 expression levels might not be predictive of response to certain CD19‐directed therapies and support previous preliminary findings that Lonca can be sequenced before or after CD19‐directed CAR‐T therapy [18, 19].

One limitation of this study is the number of patients with low CD19 expression; therefore, these results should be interpreted with caution. Additional patient samples could be useful to confirm the results of these analyses.

This work successfully integrated clinical observations and preclinical histopathology in relevant B‐cell NHL cell lines with a comprehensive QSP model that included pharmacokinetic disposition, ADC trafficking into the tumour, and translated cytotoxicity and objective tumour response. Mechanistic details of the QSP model are planned to be reported in a separate communication.

Our findings indicate that Lonca may be an effective treatment option for patients with R/R DLBCL even in patients with low levels of CD19 expression. These results suggest that CD19 IHC is perhaps not sensitive enough to be used for selecting patients for Lonca treatment. These findings also serve as a basis for future studies to address the sequencing of CD19‐targeted agents, which is a crucial topic for the scientific community involved in the treatment of patients with R/R DLBCL.

## AUTHOR CONTRIBUTIONS

PFC, MH, CC‐S, FZ, DC, SP, KH, YW and JB designed the clinical study and interpreted clinical data. MN, EJ, KU, TK, FZ, DC, KH, YW and JB contributed to the statistical analysis. All authors interpreted data, contributed critical content to the manuscript and approved its final version.

## CONFLICT OF INTEREST STATEMENT

PFC was on the advisory board for ADC Therapeutics SA, Amgen, BeiGene, Bristol Myers Squibb (BMS), Genentech, Kite, MEI Pharmaceuticals and Novartis and received research funding from AbbVie, ADC Therapeutics SA, and Genentech. MH has been a consultant with AbbVie, ADC Therapeutics SA, BMS, Caribou, CRISPR, Gamida Cell, Genmab, Incyte Corporation, Kadmon, Kite, Legend Biotech, MorphoSys, Novartis, Omeros and Seagen; received research funding from ADC Therapeutics SA and Spectrum Pharmaceuticals; has speakers bureau memberships for ADC Therapeutics SA, AstraZeneca, BeiGene, Kite, and Sanofi Genzyme and participated on data monitoring committees for Genentech and Myeloid Therapeutics. CC‐S has been a consultant with Sanofi; has been on the board of directors, speakers bureau or advisory committee for ADC Therapeutics SA, BMS, Celgene, Karyopharm, Roche and Sanofi; has received research funding from ADC Therapeutics SA, Roche and Sanofi and has received honoraria from ADC Therapeutics SA, AstraZeneca, BMS, Incyte, Janssen Oncology, and Takeda. MN is currently employed at Boehringer Ingelheim Pharmaceuticals, Inc. EJ was an employee of Metrum Research Group at the time of the study. KU and TK are currently employed at Metrum Research Group. FZ and YW were employees of ADC Therapeutics SA, a publicaly traded company, at the time of the study and may hold stock. DC, SP and JB are currently employed and current stockholders at ADC Therapeutics SA, a publicly traded company. KH is currently employed and a current stockholder at ADC Therapeutics (UK) Ltd, a publicly traded company.

### ETHICS STATEMENT

The authors have confirmed an ethical approval statement is not needed for this submission.

## CLINICAL TRIAL REGISTRATION

The authors have confirmed clinical trial registration is not needed for this submission.

## PATIENT CONSENT STATEMENT

The authors have confirmed a patient consent statement is not needed for this submission.

## Supporting information

Supporting Information

## Data Availability

The study protocol was published previously. Summary data for NCT03589469 are posted at ClinicalTrials.gov as required. Proposals requesting original participant data collected for the study can be sent to Joe.Boni@adctherapeutics.com.
