# Supplementary material for: In relapsed or refractory diffuse large B‐cell lymphoma, CD19 expression by immunohistochemistry alone is not a predictor of response to loncastuximab tesirine
Source: EJHaem. 2023 Nov 30;5(1):76–83. doi: 10.1002/jha2.816 (PMC10887233; doi:10.1002/jha2.816)
Supplement: Supplementary file 1 — Supporting Information [file JHA2-5-76-s001.pdf]

In relapsed or refractory diffuse large B-cell lymphoma, CD19 expression by immunohistochemistry alone is not a predictor of response to loncastuximab tesirine

Running title: CD19, QSP Modelling, and Response to Lonca

Paolo F. Caimi[1], Mehdi Hamadani[2], Carmelo Carlo-Stella[3], Masoud Nickaeen[4], Eric Jordie[4], Kiersten Utsey[4], Tim Knab[4], Francesca Zammarchi[5], Danilo Cucchi[5], Serafino Pantano[6], Karin Havenith[5], Ying Wang[7], Joseph Boni[7]

[1]Blood and Marrow Transplant Program, Taussig Cancer Institute, Cleveland Clinic Foundation, Cleveland, OH, USA; [2]Division of Hematology and Oncology, BMT & Cellular Therapy Program, Medical College of Wisconsin, Milwaukee, WI, USA; [3]Department of Biomedical Sciences, Humanitas University and Department of Hematology and Oncology, IRCCS Humanitas Research Hospital, Milan, Italy; [4]Metrum Research Group, Simsbury, CT, USA; [5]ADC Therapeutics (United Kingdom) Ltd, London, UK; [6]ADC Therapeutics SA, Épalinges, Switzerland; [7]ADC Therapeutics, Murray Hill, NJ, USA

Correspondence: Joseph Boni, PhD

ADC Therapeutics America, Inc.

430 Mountain Avenue, Suite 404

Murray Hill, NJ 07974 USA

E-mail: Joe.Boni@adctherapeutics.com

Supplemental Table 1. Prior systemic anti-cancer therapy, radiotherapy, surgery and stem cell transplant (all-treated population).

	150 ug/kg (*N* = 145)
Prior systemic therapies* [n(%)]	
2 prior lines	63 (43.4)
3 prior lines	34 (23.4)
>3 prior lines	48 (33.1)
Number of prior systemic therapies*	
N	145
Mean	3.1
Std	1.29
Median	3.0
Min, max	2, 7
Any prior radiotherapy [n(%)]	
Yes	53 (36.6)
No	92 (63.4)
Any prior surgery [n(%)]	
Yes	27 (18.6)
No	118 (81.4)
Any prior stem cell transplant [n(%)]	
Yes	24 (16.6)
No	121 (83.4)
Type of prior stem cell transplant [n(%)]	
Allogeneic	2 (1.4)
Autologous	21 (14.5)
Both	1 (0.7)
First line prior systemic response group [n(%)]	
Relapse	99 (68.3)
Refractory	29 (20.0)
Other	17 (11.7)
Last line prior systemic response group** [n(%)]	
Relapse	43 (29.7)
Refractory	89 (61.4)
Other	13 (9.0)

Note: *Prior stem cell transplant is included. For patients who received an autologous transplant, the mobilization regimen was considered a line of therapy if it was chemotherapy-based and distinct from the other previous lines of treatment. **If SCT is the most recent line, the variable is defined as response to the therapy immediately preceding SCT.

Supplementary Table 2. Response assessment of Hodgkin and non-Hodgkin lymphoma (Lugano Classification).

Response/site	PET-CT–based response	CT-based response
**Complete**	**Complete metabolic response**	**Complete radiologic response** (all of the following)
Lymph nodes and extralymphatic sites	• Score 1, 2, or 3* with or without a residual mass on 5PS** Note: Uptake may be greater than normal mediastinum and/or liver in Waldeyer's ring or extranodal sites with high physiologic uptake or with activation within spleen or marrow (e.g., with chemotherapy or myeloid colony-stimulating factors). In this circumstance, complete metabolic response may be inferred if uptake at sites of initial involvement is no greater than surrounding normal tissue even if the tissue has high physiologic uptake	• Target nodes/nodal masses must regress to ≤1.5 cm in LD • No extralymphatic sites of disease
Nonmeasured lesion	• Not applicable	• Absent
Organ enlargement	• Not applicable	• Regress to normal
New lesions	• None	• None
Bone marrow	• No evidence of FDG-avid disease in marrow	• Normal by morphology; if indeterminate, IHC negative
**Partial**	**Partial metabolic response**	**Partial remission** (all of the following)
Lymph nodes and extralymphatic sites	• Score 4 or 5** with reduced uptake compared with baseline and residual mass(es) of any size • At interim, these findings suggest responding disease • At end of treatment, these findings indicate residual disease	• ≥50% decrease in SPD of up to 6 target measurable nodes and extranodal sites • When a lesion is too small to measure on CT, assign 5 × 5 mm as the default value • When no longer visible, 0 × 0 mm • For a node >5 × 5 mm, but smaller than normal, use actual measurement for calculation
Nonmeasured lesion	• Not applicable	• Absent/normal, regressed, but no increase
Organ enlargement	• Not applicable	• Spleen must have regressed by >50% in length beyond normal
New lesions	• None	• None

Response/site	PET-CT–based response	CT-based response
Bone marrow	• Residual uptake higher than uptake in normal marrow but reduced compared with baseline (diffuse uptake compatible with reactive changes from chemotherapy allowed). If there are persistent focal changes in the marrow in the context of a nodal response, consideration should be given for further evaluation with MRI or biopsy or an interval scan	• Not applicable
**No response or stable disease**	**No metabolic response**	**Stable disease**
Target nodes/nodal masses, extranodal lesions	• Score 4 or 5 with no significant change in FDG update from baseline at interim or end of treatment	• <50% decrease from baseline in SPD of up to 6 dominant, measurable nodes and extranodal sites; no criteria for progressive disease are met
Nonmeasured lesions	• Not applicable	• No increase consistent with progression
Organ enlargement	• Not applicable	• No increase consistent with progression
New lesions	• None	• None
Bone marrow	• No change from baseline	• Not applicable
**Progressive disease**	**Progressive metabolic disease**	**Progressive disease** (requires at least 1 of the following)
Individual target nodes/nodal masses	• Score 4 or 5 with an increase in intensity of uptake from baseline and/or	• PPD progression
Extranodal lesions	• New FDG-avid foci consistent with lymphoma at interim or end of treatment assessment	An individual node/lesion must be abnormal with the following:<br>• LDi > 1.5 cm and<br>• Increase by ≥50% from PPD nadir and<br>• An increase in LDi or SDi from nadir<br>0.5 cm for lesions ≤ 2 cm<br>1.0 cm for lesions > 2 cm<br>• In the setting of splenomegaly, the splenic length must increase by > 50% of the extent of its prior increase beyond baseline (e.g., a 15-cm spleen must increase to > 16 cm). If no prior splenomegaly, must increase by at least 2 cm from baseline<br>• New or recurrent splenomegaly

Response/site	PET-CT–based response	CT-based response
Nonmeasured lesions	• None	• New or clear progression of preexisting nonmeasured lesions
New lesions	• New FDG-avid foci consistent with lymphoma rather than another etiology (e.g., infection, inflammation). If uncertain regarding etiology of new lesions, biopsy or interval scan may be considered	• Regrowth of previously resolved lesions<br>• A new node > 1.5 cm in any axis<br>• A new extranodal site > 1.0 cm in any axis; if < 1.0 cm in any axis, its presence must be unequivocal and must be attributable to lymphoma<br>• Assessable disease of any size unequivocally attributable to lymphoma
Bone marrow	• New or recurrent FDG-avid foci	• New or recurrent involvement

Abbreviations: 5PS, 5-point scale; CT, computed tomography; FDG, fluorodeoxyglucose; IHC, immunohistochemistry; LDi, longest transverse diameter of a lesion; MRI, magnetic resonance imaging; PET, positron emission tomography; PPD, cross product of the LDi and perpendicular diameter; SDi, shortest axis perpendicular to the LDi; SPD, sum of the product of the perpendicular diameters for multiple lesions.

*A score of 3 in many patients indicates a good prognosis with standard treatment, especially if at the time of an interim scan. However, in trials involving PET where de-escalation is investigated, it may be preferable to consider a score of 3 as inadequate response (to avoid undertreatment). Measured dominant lesions: up to six of the largest dominant nodes, nodal masses and extranodal lesions selected to be clearly measurable in two diameters. Nodes should preferably be from disparate regions of the body and should include, where applicable, mediastinal and retroperitoneal areas. Non-nodal lesions include those in solid organs (e.g., liver, spleen, kidneys and lungs), GI involvement, cutaneous lesions or those noted on palpation. Nonmeasured lesions: any disease not selected as measured; dominant disease and truly assessable disease should be considered not measured. These sites include any nodes, nodal masses and extranodal sites not selected as dominant or measurable or that do not meet the requirements for measurability but are still considered abnormal, as well as truly assessable disease, which is any site of suspected disease that would be difficult to follow quantitatively with measurement, including pleural effusions, ascites, bone lesions, leptomeningeal disease, abdominal masses and other lesions that cannot be confirmed and followed by imaging. In Waldeyer's ring or in extranodal sites (e.g., GI tract, liver and bone marrow), FDG uptake may be greater than in the mediastinum with complete metabolic response but should be no higher than surrounding normal physiologic uptake (e.g., with marrow activation as a result of chemotherapy or myeloid growth factors).

** PET 5PS: 1, no uptake above background; 2, uptake ≤ mediastinum; 3, uptake > mediastinum but ≤ liver; 4, uptake moderately > liver; 5, uptake markedly higher than liver and/or new lesions; X, new areas of uptake unlikely to be related to lymphoma.

Reference: Cheson BD, Fisher RI, Barrington SF, et al. Recommendations for initial evaluation, staging and response assessment of Hodgkin and non-Hodgkin lymphoma: The Lugano classification. *J Clin Oncol* 2014;32(27):3059-3067.

Supplementary Table 3. Quantitative systems pharmacology model parameters, values and source information. Unless otherwise noted, the model structure, equations and parameterization are as found in Jones et al. 2019 (Jones HM, Zhang Z, Jasper P, Luo H, Avery LB, King LE, *et al*. A physiologically-based pharmacokinetic model for the prediction of monoclonal antibody pharmacokinetics from in vitro data. *CPT Pharmacometrics Syst Pharmacol.* 2019; **8**[10]: 738–47. doi: 10.1002/psp4.12461).

**Parameter**	**Definition**	**Value(s)**	**Unit**	**Source**
FcRn_Conc	FcRn expression level	22.0465 - 44.093	uM	Upper bound from https://ascpt.onlinelibrary.wiley.com/doi/10.1002/psp4.12461; HA patients simulated with a lower levels
mu	CD19+ tumor cell growth rate	0.0065 - 0.0195	1/h	Lower bound assumes a 107 h doubling time (typical value from https://link.springer.com/article/10.1016/j.bulm.2004.06.007); DH/TH patients simulated with faster rates
mu_n	CD19-/low tumor cell growth rate	0.0065 - 0.0195	1/h	Lower bound assumes a 107h doubling time (typical value from https://link.springer.com/article/10.1016/j.bulm.2004.06.007); DH/TH patients simulated with faster rates
cd19_exp_tum	Ratio of CD19+ tumor cells to total tumor cells in tumor tissue	0.01 - 1.0	unitless	Informed by IHC data, default value was 0.9
Rn0	Number of CD19 antigens per CD19-/low cell	1 - 10	unitless	Values chosen to be low/undetectable
R0	Number of CD19 antigens per CD19+ cell	100 - 21000	unitless	Median value taken from Fig 4. in https://acsjournals.onlinelibrary.wiley.com/doi/10.1002/cncr.33796
K_D	CD19-ADC dissociation constant	2.06E-04	uM	Fit to data from https://onlinelibrary.wiley.com/doi/full/10.1111/j.1365-2141.2007.06883.x?sid=nlm%3Apubmed
k_on	CD19-ADC complex formation rate constant	2934	1/uM/h	Fit to data from https://onlinelibrary.wiley.com/doi/full/10.1111/j.1365-2141.2007.06883.x?sid=nlm%3Apubmed
k_e	Endocytosis rate constant	0.165	1/h	Fit to data from https://onlinelibrary.wiley.com/doi/full/10.1111/j.1365-2141.2007.06883.x?sid=nlm%3Apubmed
k_deg	Rate constant for lysosomal degradation and intracellular payload release	1.137	1/h	Fit to data from https://onlinelibrary.wiley.com/doi/full/10.1111/j.1365-2141.2007.06883.x?sid=nlm%3Apubmed
k_out	Rate constant for	0.05	1/h	Typical value from https://jpet.aspetjournals.org/content/374/1/184

	payload leaving the cell via diffusion			
k_in	Rate constant of payload entering the cell via diffusion	0.5	1/h	Typical value from https://jpet.aspetjournals.org/content/374/1/184
r	Tumor cell radius	5.00E-06	um	Internal data
PBD_IC50	Payload cytotoxicity IC50 value for CD19+ cells	1.59E-04	uM	Internal data
PBD_IC50_n	Payload cytotocicity IC50 for CD19-/low cells	2.55E-04	nM	Internal data
DAR	Drug-antibody ratio	2.3	unitless	From https://ashpublications.org/blood/article/131/10/1094/36428/ADCT-402-a-PBD-dimer-containing-antibody-drug
k_death	Death rate of tumor cells	0.051	1/h	Fit to internal tumor growth inhibition data
k_trans	Death rate of transit cells	0.001	1/h	Fit to internal tumor growth inhibition data
CD21Factor	Ratio of CD21 to CD19 antigens on tumor cell surface	2.515	unitless	From https://onlinelibrary.wiley.com/doi/10.1111/j.1365-2141.2007.06883.x
k_on21	CD21-CD19-ADC complex formation rate constant	9.16E-05	1/(#/cell)/h	Fit to data from https://onlinelibrary.wiley.com/doi/full/10.1111/j.1365-2141.2007.06883.x?sid=nlm%3Apubmed
k_off21	CD21-CD19-ADC complex dissociation rate constant	3.24E-05	1/h	Fit to data from https://onlinelibrary.wiley.com/doi/full/10.1111/j.1365-2141.2007.06883.x?sid=nlm%3Apubmed
k_el_payload	Elimination rate constant of the payload in the extracellular space	0.693	1/h	Assumed half-life of payload in plasma is ~1h https://pubmed.ncbi.nlm.nih.gov/29992976/

ACSINS_score	AC-SINS score of the mAb	6	unitless	Fit to internal data

Variable	Definition	Unit
R_nm	Number of free surface receptors (CD19) per CD19+ tumor cell	#/cell
C_nm	Number of ADC-CD19 complexes per surface of CD19+ tumor cell	#/cell
I_nm	Number of internalized ADC-CD19 complexes per CD19+ tumor cell	#/cell
De_nm	Number of degraded ADCs per CD19+ tumor cell	#/cell
R21_nm	Number of free surface receptors (CD21) per CD19+ tumor cell	#/cell
CRR21_xnm	Number of CD19-CD21-ADC complexes per CD19+ tumor cell	#/cell
Rn_xnm	Number of free surface receptors (CD19) per CD19-/low tumor cell	#/cell
Cn_xnm	Number of ADC-CD19 complexes per surface of CD19-/low tumor cell	#/cell
In_xnm	Number of internalized ADC-CD19 complexes per CD19-/low tumor cell	#/cell
Den_xnm	Number of degraded ADCs per CD19-/low tumor cell	#/cell
R21n_xnm	Number of free surface receptors (CD21) per CD19-/low tumor cell	#/cell
CRR21n_xnm	Number of CD19-CD21–ADC complexes per CD19-/low tumor cell	#/cell
N_nm	Number of CD19+ tumor cells	#
Nn_nm	Number of CD19-/low tumor cells	#
Dm_nm	Concentation of payload in the tumor interstitial space	uM
Nd_xnm	Number of CD19+ transit cells	#
Nnd_xnm	Number of CD19-/low transit cells	#
Tumor_Volume	Tumor volume (calculated from sum of tumor cells, assuming tumor has cellularity of 0.375)	L

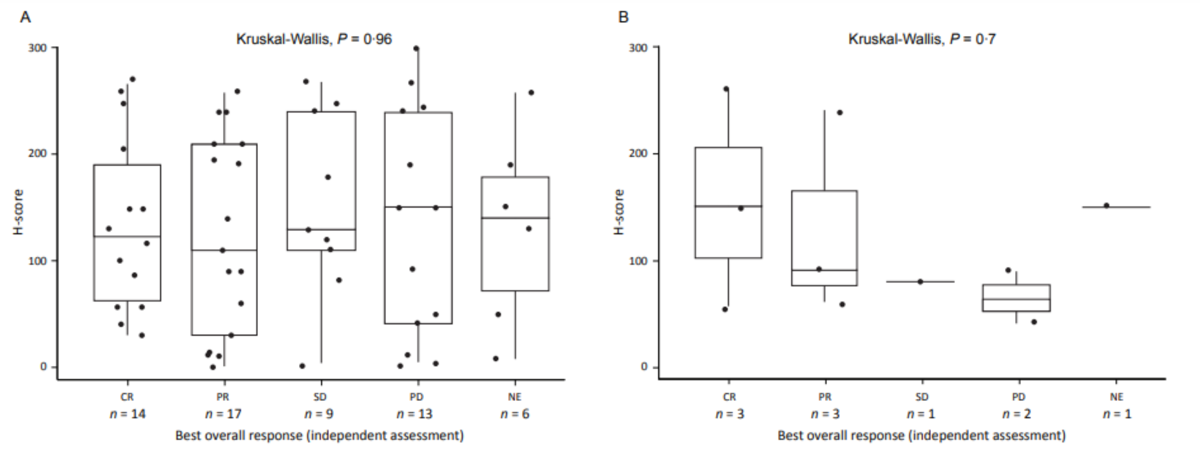

Supplementary Fig S1. Baseline tumour CD19-positive H-score by response category (independent assessment). (A) Immunohistochemistry analysis of biopsy samples from patients enrolled in the LOTIS-2 study, with biopsies that were collected after the last systemic therapy and before Lonca treatment (*n* = 59) and (B) from patients after CAR-T therapy (*n* = 10). CAR-T, chimeric antigen receptor T-cell; CR, complete response; H-score, histoscore; NE, not evaluable; PD, progressive disease; PR, partial response; SD, stable disease.

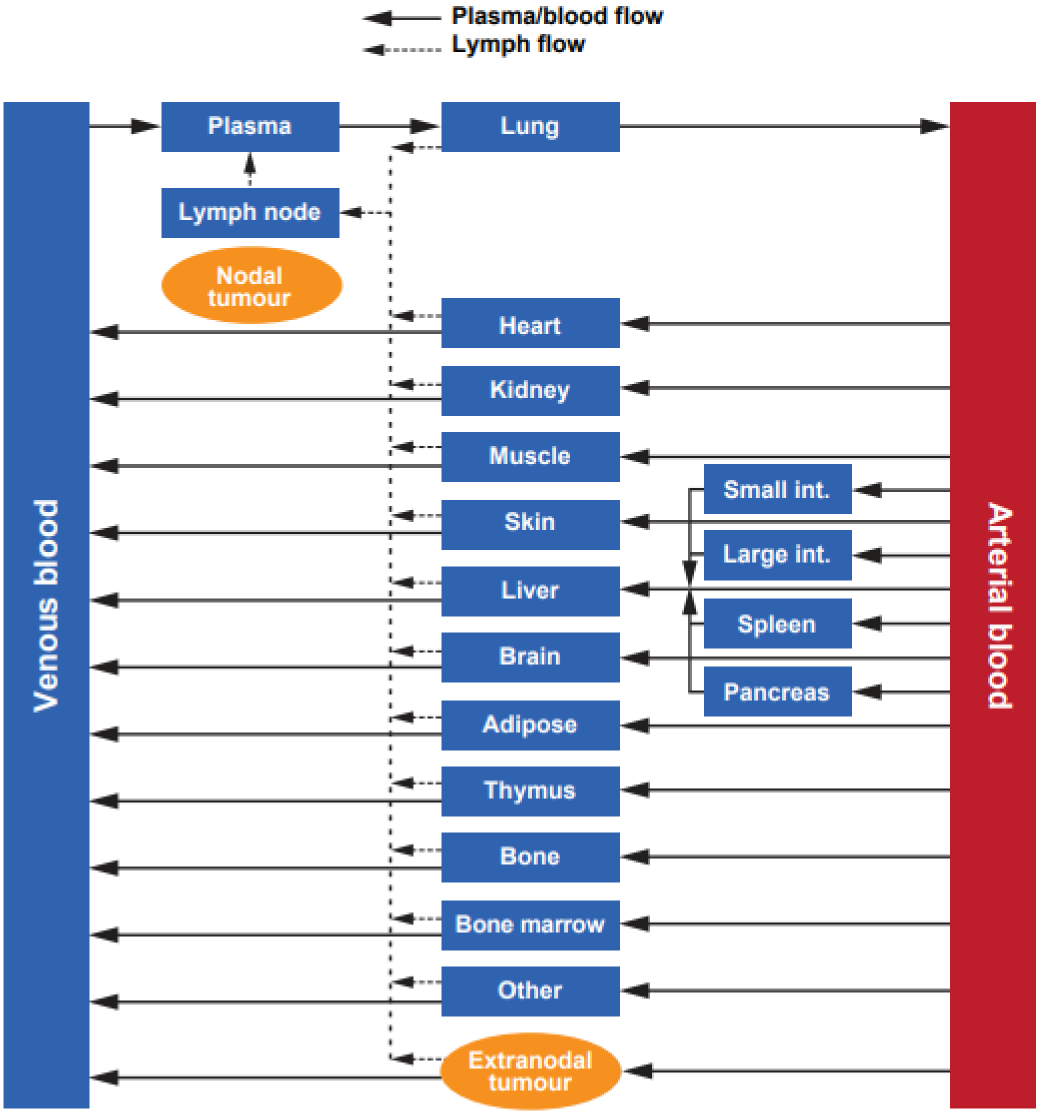

Supplementary Fig S2. Quantitative systems pharmacology model schematic. This model has been extended to include nodal and extranodal tumour compartments. (Adapted from Jones HM, Zhang Z, Jasper P, Luo H, Avery LB, King LE, *et al*. A physiologically-based pharmacokinetic model for the prediction of monoclonal antibody pharmacokinetics from in vitro data. *CPT Pharmacometrics Syst Pharmacol.* 2019; **8**[10]: 738–47. doi: 10.1002/psp4.12461)

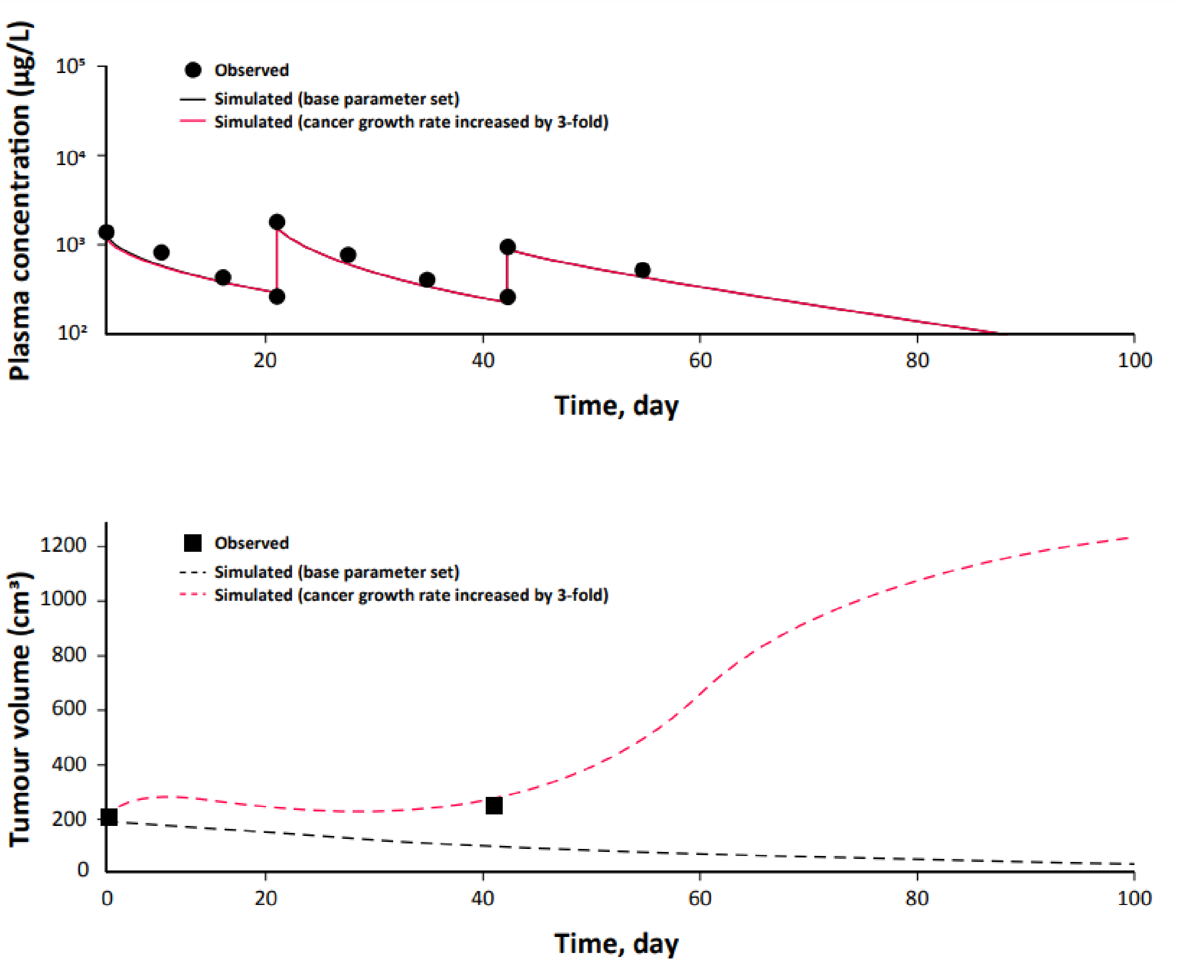

Supplementary Fig 3. Quantitative systems pharmacology modelling indicated that the faster growth rate of double-hit lymphomas explained the observed lack of response to Lonca, despite high CD19 positivity as measured by IHC. The example patient had a body weight of 122 kg and had double-hit lymphoma; the IHC of the biopsy showed that 95% of cells were CD19 positive. IHC, immunohistochemistry; Lonca, loncastuximab tesirine-lpyl.

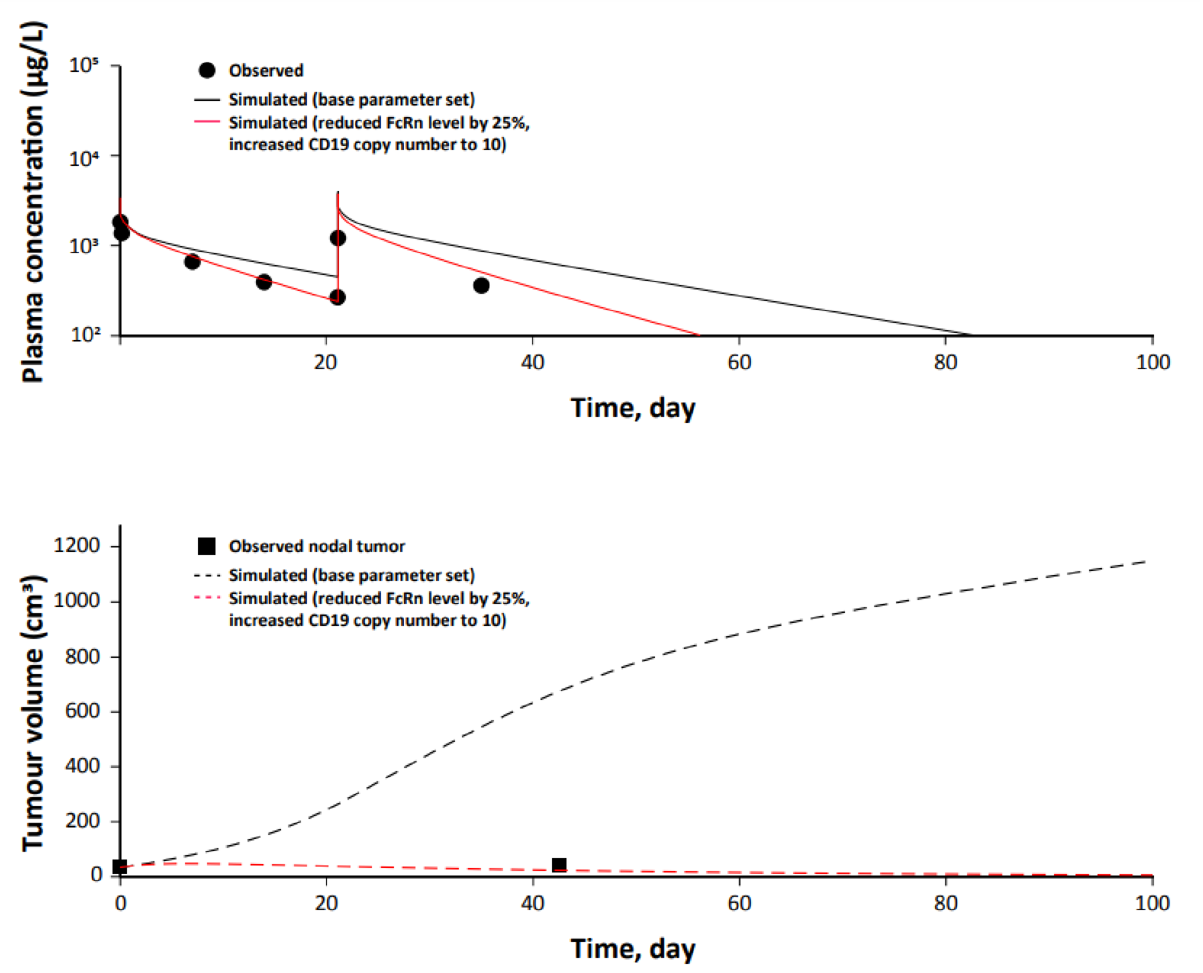

Supplementary Fig 4. Quantitative systems pharmacology modelling indicated that patients with hypoalbuminemia had enhanced clearance and reduced Lonca exposure, as assessed through a reduction in FcRn expression. The example patient had a body weight of 66 kg and had hypoalbuminemia; the IHC of the biopsy showed that 1% of cells were CD19 positive. FcRn, neonatal Fc receptor; IHC, immunohistochemistry; Lonca, loncastuximab tesirine-lpyl.
